# Subnuclear gene positioning through lamina association affects copper tolerance

**DOI:** 10.1038/s41467-020-19621-z

**Published:** 2020-11-20

**Authors:** Yuki Sakamoto, Mayuko Sato, Yoshikatsu Sato, Akihito Harada, Takamasa Suzuki, Chieko Goto, Kentaro Tamura, Kiminori Toyooka, Hiroshi Kimura, Yasuyuki Ohkawa, Ikuko Hara-Nishimura, Shingo Takagi, Sachihiro Matsunaga

**Affiliations:** 1 Imaging Frontier Center, Organization for Research Advancement, Tokyo University of Science, 2641 Yamazaki, Noda, Chiba 278-8510 Japan; 2 Department of Biological Sciences, Graduate School of Science, Osaka University, 1-1 Machikaneyama-cho, Toyonaka, Osaka 560-0043 Japan; 3 RIKEN Center for Sustainable Resource Science, Yokohama, 230-0045 Japan; 4 Institute of Transformative Bio-Molecules, Nagoya University, Chikusa, Nagoya 464-8601 Japan; 5 Division of Transcriptomics, Medical Institute of Bioregulation, Kyushu University, 3-1-1 Maidashi, Higashi, Fukuoka 812-0054 Japan; 6 College of Bioscience and Biotechnology, Chubu University, Kasugai, 487-8501 Japan; 7 Graduate School of Agricultural and Life Sciences, The University of Tokyo, 1-1-1 Yayoi, Bunkyo-ku, Tokyo 113-8657 Japan; 8 School of Food and Nutritional Sciences, University of Shizuoka, Shizuoka, 422-8526 Japan; 9 Graduate School of Bioscience and Biotechnology, Tokyo Institute of Technology, Yokohama, 226-8501 Japan; 10 Faculty of Science and Engineering, Konan University, Kobe, 658-8501 Japan; 11 Department of Applied Biological Science, Faculty of Science and Technology, Tokyo University of Science, 2641 Yamazaki, Noda, Chiba 278-8510 Japan; 12 Department of Integrated Biosciences, Graduate School of Frontier Sciences, The University of Tokyo, 5-1-5 Kashiwanoha, Kashiwa, Chiba 277-8562 Japan; 13 Present Address: Graduate School of Science, Kobe University, Kobe, 657-8501 Japan

**Keywords:** Developmental biology, Plant sciences

## Abstract

The nuclear lamina plays an important role in the regulation of chromatin organization and gene positioning in animals. CROWDED NUCLEI (CRWN) is a strong candidate for the plant nuclear lamina protein in *Arabidopsis thaliana* but its biological function was largely unknown. Here, we show that CRWNs localize at the nuclear lamina and build the meshwork structure. Fluorescence in situ hybridization and RNA-seq analyses revealed that CRWNs regulate chromatin distribution and gene expression. More than 2000 differentially expressed genes were identified in the *crwn1crwn4* double mutant. Copper-associated (*CA*) genes that form a gene cluster on chromosome 5 were among the downregulated genes in the double mutant exhibiting low tolerance to excess copper. Our analyses showed this low tolerance to copper was associated with the suppression of *CA* gene expression and that CRWN1 interacts with the *CA* gene locus, enabling the locus to localize at the nuclear lamina under excess copper conditions.

## Abstract (web summary)

The nuclear lamina regulates chromatin organization and gene positioning. Here the authors show that CROWDED NUCLEI proteins contribute to the meshwork lamina structure in *Arabidopsis* nuclei and regulate copper tolerance by promoting lamina association and expression of copper response genes.

## Introduction

Chromatin does not drift in the nucleoplasm but instead associates with inner nuclear membranes, the nuclear lamina, nuclear pores, the nucleolus, and nuclear bodies, all of which are components responsible for the non-random spatial positioning of genomic loci^1,2^. Several studies have suggested that gene positioning in the three-dimensional subnuclear space affects transcriptional activity^3^. In budding yeast, several genes are located at the nuclear periphery when they are activated^4–7^. In many types of mammalian cells, inactivated genes are positioned at the nuclear periphery, while activated genes are localized around the center of the nucleoplasm^8^. However, the effects of gene positioning on transcription in plants are unknown.

As the location of a gene at the nuclear periphery can affect its expression, the nuclear lamina plays important roles as a platform for gene localization and/or expression. The nuclear lamina, a meshwork structure beneath the inner nuclear membrane, has been observed in animal and plant cells^9,10^. In metazoans, the nuclear lamina is mainly composed of lamins, which interact with each other via their central rod domain containing four coiled-coil regions to form intermediate filaments and a meshwork structure that mechanically supports the nuclear membrane^11,12^. Lamins can directly and/or indirectly bind to chromatin to control the distribution and positioning of chromatin and gene activity. Nuclear lamina-associated domains (LADs) have been identified and mapped in animal cells by chromatin immunoprecipitation (ChIP) and DNA adenine methyltransferase identification (Dam-ID) methods. The LADs include low-expression genes, gene-free genomic regions, and repeat sequences such as pericentromeres, and are enriched in histone modifications for heterochromatin^13^.

Although lamin homologs have not been found in plant genomes, the meshwork structure has been observed in the nuclear lamina by electron microscopy, which implies that unidentified proteins make up the nuclear lamina structure^10,14^. The crowded nuclei (CRWN) proteins are strong candidates for functional lamin-like proteins. CRWNs were first identified as nuclear matrix constituent proteins (NMCPs) from carrot and are widely conserved in land plants^15^. Like lamins, CRWNs contain a long coiled-coil region at their N-terminal. *Arabidopsis thaliana* harbors four CRWN homologs that are phylogenetically separated into two groups: the NMCP1 group, including CRWN1, CRWN2, and CRWN3; and the NMCP2 group including CRWN4^16,17^. In previous studies, CRWNs were detected in the nuclear lamina fraction and localized at the nuclear periphery^18^. Single or double *crwn* mutants have small and abnormally shaped nuclei^16,18^. Double mutants and some triple mutants have a dwarf phenotype, and some triple and quadruple mutants are lethal^19^. CRWN1 and CRWN3 play roles in seed dormancy by inhibiting the degradation of ABI5^20^. CRWNs are also involved in resistance against virulent bacterial pathogens^21^. CRWN1 is degraded in a proteasome-mediated manner in response to pathogens, resulting in activation of the plant immune response gene pathogenesis-related protein 1 (PR1).

Here, we report that CRWNs form the meshwork structure at the nuclear lamina. We demonstrate that the upregulation of copper-associated genes is required for copper tolerance and is inhibited in *crwn1crwn4* mutants with low copper tolerance. The gene locus interacts with CRWN1 and is anchored to the nuclear periphery, which activates gene expression, only under excess copper conditions.

## Results

### CRWNs specifically localize and build a meshwork structure at the nuclear periphery

**Fig. 1:**
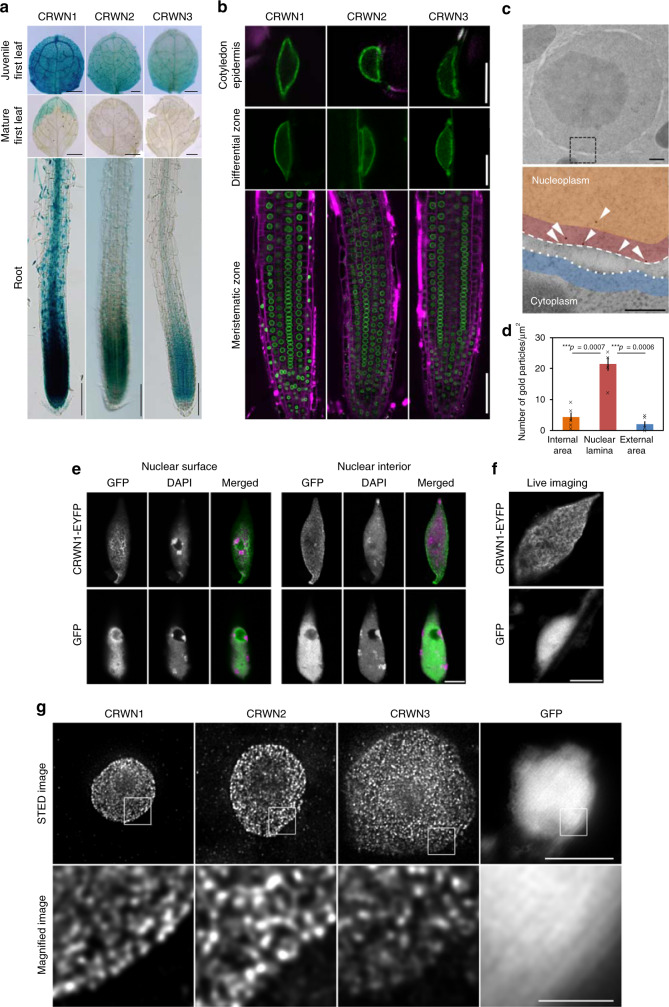
CRWN expression and localization patterns in *Arabidopsis thaliana*. **a** β-Glucuronidase (GUS) signals in young first leaves (8-day-old plant), adult first leaves (14-day-old plant), and primary roots of *pCRWN1*::CRWN1-GUS, *pCRWN2*::CRWN2-GUS, and *pCRWN3*::CRWN3-GUS. Scale bars = 200 µm (top), 1 mm (middle), and 100 µm (bottom). **b** Confocal fluorescence images of cotyledons and roots showing *pCRWN1*::CRWN1-EYFP in *crwn1*, *pCRWN2*::CRWN2-EYFP in *crwn2*, and *pCRWN3*::CRWN3-sGFP in *crwn3*. EYFP and sGFP signals are green and propidium iodide signals are magenta. Scale bars = 10 µm (top and middle) and 50 µm (bottom). **c** Transmission electron micrograph of the nucleus in a root meristematic cell. Lower panel shows a magnified image of the broken square in the upper panel. White arrowheads mark gold particle signals. Dotted and broken lines indicate outer and inner nuclear membranes, respectively. Red, orange, and blue areas indicate the nuclear lamina, internal, and external areas, respectively. Scale bars = 200 nm. **d** Number of gold particles in each area. Data are mean ± SEM. Significance was determined using unpaired two-sided *t*-test (*n* = 5 individual nucleus). Each data point represents a cross mark. **e** Immunofluorescence images of nuclear surface and nuclear interior. Immunostaining signals are green and DAPI signals are magenta in the merged panel. Scale bar = 5 µm. **f** Confocal fluorescence images of root epidermal cells in the elongation zone. Scale bar = 5 µm. **g** STED images of nuclei isolated from 7-day-old seedlings. Lower panels are magnified images of the area indicated by the small square in the upper panels. Scale bars = 5 µm (top) and 1 µm (bottom).

In contrast to previous studies in which CRWN1–4 proteins were expressed under the control of a strong 35S promoter, we here wanted to test the expression and localization patterns upon expression under the control of the native promoter. Towards this goal, we prepared *pCRWN*::CRWN-GUS in the wild type (WT) and *pCRWN*::CRWN-EYFP and -sGFP in each *crwn* mutant (Fig. 1a, b). EYFP(S65G/V68U/S72A/T203Y) and sGFP(S65T) are variants of the green fluorescent protein^22,23^. CRWN-GUS, -EYFP, and -sGFP were driven by the native promoter (defined as the 2-kbp sequence upstream from the start codon). Although we constructed *pCRWN4*::CRWN4-GUS in WT, we did not detect any signals (Supplementary Fig. 1).

CRWN1–3 showed similar expression and localization patterns (Fig. 1a, b). CRWN1–3 were strongly expressed in the root apical meristem and young leaves but rarely expressed in mature leaves (Fig. 1a). CRWN1–3 were specifically localized at the periphery in both spherical nuclei and elongated nuclei (Fig. 1b). To reveal whether CRWNs localized at the nuclear lamina area, *pCRWN1*::CRWN1-EYFP in *crwn1* was observed by immunoelectron microscopy using an anti-GFP antibody and a gold-conjugated secondary antibody (Fig. 1c). The majority of the CRWN1 signals appeared to be observed in the nuclear lamina (Fig. 1c). To quantify the signals, the nucleus was classified into three areas: the nuclear lamina, internal, and external area. The nuclear lamina area and the external area were the parts within 100 nm of the inner and outer nuclear membranes, respectively. The internal area was the inner region further inside from the nuclear lamina area. Gold particles were more abundant at the nuclear lamina area than at other areas, indicating that CRWN1 specifically localized at the nuclear periphery, suggesting that it could be a major constituent of the plant nuclear lamina (Fig. 1d).

Next, to investigate the distribution of CRWN1 at the nuclear lamina, *pCRWN1*::CRWN1-EYFP was visualized in isolated nuclei by immunofluorescence staining using an anti-GFP antibody. CRWN1 showed the meshwork pattern at the nuclear periphery (Fig. 1e). In live imaging analysis, *pCRWN1*::CRWN1-EYFP also formed a meshwork pattern at the nuclear periphery in a root epidermal cell (Fig. 1f). Furthermore, detailed distribution patterns of CRWN1–3 were observed by stimulated emission depletion (STED) microscopy, which is one of the super-resolution microscopies and provides sub-diffraction-limit imaging by using a second laser negating the emission from fluorophores located away from the centre of excitation. CRWN1–3 exhibited similar continuous structures (Fig. 1g). These results suggested that CRWN1–3 proteins built the meshwork structure at the plant nuclear lamina area.

**Fig. 2:**
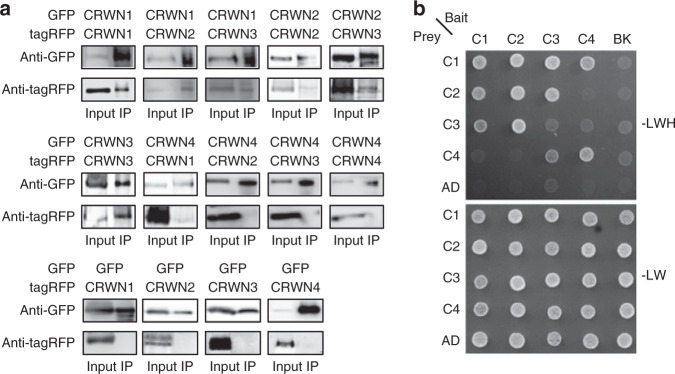
Interactions among CRWNs. **a** Analysis of the interactions among CRWNs in *Nicotiana benthamiana* leaves detected by co-immunoprecipitation assay. Data are from single representative experiments that were reproduced twice. **b** Analysis of the CRWN interactions by yeast two-hybrid assay. Growth tests of yeast expressing CRWNs on agar plates lacking leucine, tryptophan, and histidine (top), or leucine and tryptophan (bottom). Empty vectors of bait (BK) and prey (AD) served as negative controls.

To determine whether CRWNs interact with each other, which is probably required for construction of the meshwork structure, protein–protein interactions were analyzed by co-immunoprecipitation (Co-IP) assay and yeast two-hybrid assay (Y2H). Both assays indicated that CRWN1, CRWN2, and CRWN3 interacted with each other, and that CRWN1 also interacted with CRWN4 (Fig. 2a, b). However, CRWN4 could not interact with CRWN2 or CRWN3. The homomeric interaction of CRWN3 was detected by Co-IP but not by Y2H and that of CRWN4 was detected by Y2H but not by Co-IP, suggesting that the CRWN3 interaction negatively affected the reporter gene expression in yeast and the CRWN4 interaction was too weak to be detected by Co-IP. These protein interaction assays demonstrated that all CRWNs could form homo- and/or hetero-oligomers for construction of the meshwork structure.

### CRWNs regulate chromatin distribution and gene expression

**Fig. 3:**
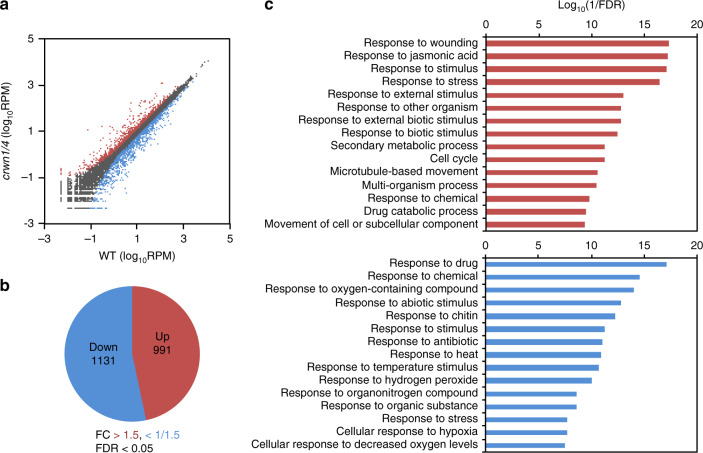
CRWNs affect gene expression pattern. **a** Scatter plots of RNA-seq data comparing mRNA abundance between WT and *crwn1crwn4*. Red marks denote mRNAs upregulated more than 1.5-fold (FDR < 0.05); blue marks denote those downregulated to <0.667-fold (FDR < 0.05); gray marks denote other mRNAs. **b** Pie chart of differentially expressed genes (DEGs). **c** Enrichment analysis of GO terms for upregulated genes (top) and downregulated genes (bottom). GO analysis according to biological process.

To explore the function of CRWNs in chromatin organization, we investigated the chromatin distribution in *crwn* single and double mutants by fluorescence in situ hybridization (FISH). The number of signals derived from 180-bp pericentromeric sequences was around 10 in WT nuclei, but significantly lower in nuclei of *crwn1*, *crwn4*, *crwn1crwn2*, and *crwn1crwn4*, suggesting that CRWNs are involved in the maintenance of chromatin structure and distribution (Supplementary Fig. 2a). Instead of a reduction of the number of signals, the size and intensity of a signal were increased in the *crwn1crwn4* (Supplementary Fig. 2b, c), suggesting that pericentromeric regions were not lost but aggregated with each other. Next, the RNA-seq analysis of 2-week-old WT and *crwn1crwn4* was performed to determine whether the gene expression pattern was also affected by the defect of CRWN1 and CRWN4. Gene expression data were normalized and compared using TCC in the R software package to identify differentially expressed genes (DEGs) between WT and *crwn1crwn4*. The DEGs were defined as those with transcript levels greater than 1.5-fold or reduced to less than 0.667-fold and a false-discovery rate (FDR) of less than 0.05. We identified 2122 DEGs (991 upregulated genes and 1131 downregulated genes in *crwn1crwn4 vs*. WT) (Fig. 3a, b). The Gene Ontology analysis revealed that, among both up- and downregulated genes, there was the enrichment of biotic and abiotic stress-responsive genes (Fig. 3c), suggesting that CRWN1 and CRWN4 mainly regulate stress response pathways.

### CRWN1 and CRWN4 regulate transcript levels of copper-associated genes

**Fig. 4:**
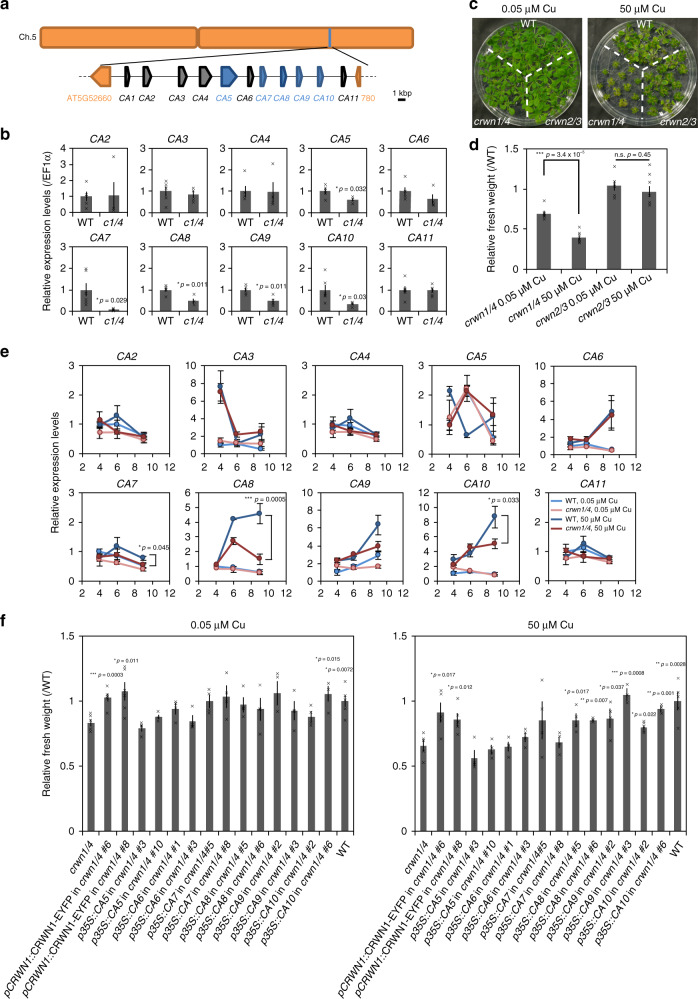
CRWNs elevate expression of *CA* genes. **a** Schematic figure of the *CA* gene locus on chromosome 5. Downregulated genes identified by qRT-PCR are shown in blue and other *CA* genes are shown in gray. **b** qRT-PCR analysis of *CA* genes in 2-week-old WT and *crwn1crwn4* plants. Data were normalized to EF1α mRNA levels and are expressed as mean ± SEM relative to the WT value (defined as 1). Significance was determined using unpaired two-sided *t*-test (*n* = 5 individual experiments). Each data point represents a cross mark. **c** WT, *crwn1crwn4*, and *crwn2crwn3* grown under normal and excess copper conditions for 2 weeks. **d** Fresh weight of 2-week-old WT, *crwn1crwn4*, and *crwn2crwn3* grown under normal (*n* = 6 individual experiments) and excess copper conditions (*n* = 8 individual experiments). Fresh weights of *crwn1crwn4* and *crwn2crwn3* were normalized against that of WT to calculate relative fresh weight and are expressed as mean ± SEM. Significance was determined using unpaired two-sided *t*-test. Each data point represents a cross mark. **e** qRT-PCR analysis of *CA* gene transcript levels in 4-, 6-, and 9-day-old WT and *crwn1crwn4* under normal and excess copper conditions. Data were normalized to EF1α mRNA levels and are expressed as mean ± SEM relative to the value of 4-day-old WT under normal condition (defined as 1). Significance was determined using unpaired two-sided *t*-test (*n* ≥ 3 individual experiments). **f** Fresh weight of 2-week-old WT, *crwn1crwn4*, and *crwn1crwn4* expressing CRWN1-EYFP and *CA* genes under normal and excess copper conditions. Fresh weights of *crwn1crwn4* and *crwn1crwn4* expressing CRWN1-EYFP and *CA* genes were normalized against that of WT to calculate relative fresh weight and are expressed as mean ± SEM. Significance was determined using unpaired two-sided *t*-test (vs *crwn1crwn4*; *n* ≥ 3 individual experiments). Each data point represents a cross mark.

Three copper-associated (*CA*) genes, belonging to the family encoding heavy metal-associated proteins in plants, were among the downregulated DEGs in *crwn1crwn4*. Heavy metal-associated domains are conserved among the family members and are involved in the chelating of metal ions in the cytoplasm^24^. The 11 *CA* genes (*CA1–11*) including the three genes mentioned above are tandemly localized on the long arm of chromosome 5 (Fig. 4a and Supplementary Table 1). Quantitative RT-PCR (qRT-PCR) analyses of 2-week-old plants revealed that half of the genes in this cluster (*CA5* and *CA7–10*) were downregulated in *crwn1crwn4* compared with the levels in WT (Fig. 4b). Interestingly, the expression levels of *CA5–10* genes were unchanged in *crwn2crwn3*, exhibiting an almost normal chromatin distribution pattern (Fig. 3a and Supplementary Fig. 3). These results suggested that CRWN1 and CRWN4 but not CRWN2 and CRWN3 transcriptionally regulate *CA* genes.

### CRWNs contribute to copper tolerance by upregulating *CA* genes

Although the function of *CA* genes has not been revealed, they all contain a heavy metal-associated domain^24^. Thus, we investigated the copper tolerance of *crwn* mutants. The fresh weight of 2-week-old plants of WT, *crwn1crwn4*, and *crwn2crwn3* was measured under normal (0.05 µM Cu) and excess copper conditions (50 µM Cu). The fresh weight of *crwn1crwn4* was approximately 70% of that of WT under normal conditions, but significantly reduced to approximately 40% of that of WT under excess copper conditions (Fig. 4c, d). Thus, *crwn1crwn4* was hypersensitive to excess copper. In contrast, the fresh weight of *crwn2crwn3* under both conditions was similar to that of WT (Fig. 4c, d).

As 2-week-old plants of *crwn1crwn4* showed weak tolerance to excess copper, we investigated the transcript levels of *CA* genes at the early stage (4-, 6-, and 9-day-old seedlings) under both normal and excess copper conditions by qRT-PCR. The transcript levels of *CA6–10* were increased in response to excess copper in WT at day 9 (Fig. 4e). However, the transcript levels of *CA7, CA8*, and *CA10* were lower in *crwn1crwn4* than in WT under excess copper conditions at day 9 (Fig. 4e). The transcript level of copper/zinc superoxide dismutase (CSD) as a positive control was dramatically elevated by excess copper in WT as well as in *crwn1crwn4* (Supplementary Fig. 4). To clarify whether low expression of *CA* genes caused the weak copper tolerance of *crwn1crwn4*, we generated transformants overexpressing *CA* genes in *crwn1crwn4*. Almost all of the transgenic plants significantly overexpressed the transgenes compared with the level in the WT (Supplementary Fig. 5). Two individual lines of *p35S*::*CA10* showed similar expression levels to that of WT but significantly increased levels compared with that of the parental line *crwn1crwn4*. The fresh weight of *pCRWN1*::CRWN1-EYFP in *crwn1crwn4* (positive control) was significantly greater than that of *crwn1crwn4* under both normal and excess copper conditions, suggesting that the cause of the dwarf phenotype was knockout of *CRWN*s and that CRWN1-EYFP could complement this phenotype (Fig. 4f). The fresh weights of *crwn1crwn4* expressing *CA5–7* were similar to that of *crwn1crwn4* under both conditions. In contrast, the fresh weights of *crwn1crwn4* expressing *CA8–10* were significantly greater than that of *crwn1crwn4* only under excess copper conditions, except for *p35S*::*CA10* #6 (Fig. 4f). These results strongly suggested that the low transcript levels of *CA8, CA9*, and *CA10* resulted in the weak copper tolerance of *crwn1crwn4*.

### CRWNs alter the position of *CA* genes in the nucleus

**Fig. 5:**
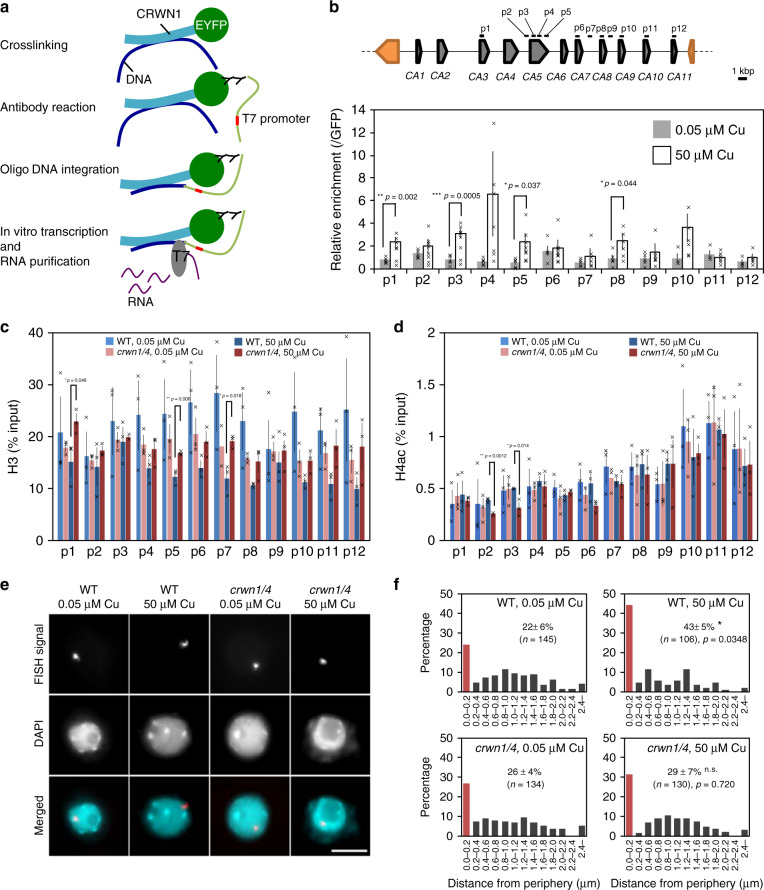
CRWNs regulate the position of the *CA* gene locus depending on copper concentration. **a** Outline of chromatin integration labeling (ChIL) method. **b** ChIL-qPCR analyses using 12 primer pairs described as p1 to p12. Top image indicates the position of primer pairs at the *CA* gene locus. Data were normalized to ChILed DNA levels in GFP control and are expressed as mean ± SEM. Significance was determined using unpaired two-sided *t*-test (*n* ≥ 4 individual experiments). Each data point represents a cross mark. **c**, **d** ChIP assay for histone H3 (**c**) and H4 acetylation (**d**) in the *CA* gene locus under normal and excess copper conditions. Data are expressed as mean ± SEM. Significance was determined using unpaired two-sided *t*-test (*n* = 3 individual experiments). Each data point represents a cross mark. **e** Visualization of the *CA* gene locus in the nucleus by padlock FISH. In the merged panel, FISH signals are red and DAPI signals are cyan. Scale bar = 5 µm. Data are from single representative experiments that were reproduced three times. **f** The distance between the *CA* gene locus and the nuclear edge was measured. The red bars in the histogram represent the nuclear periphery including the region 0.0 to 0.2 μm from the nuclear edge. The average percentage of the *CA* gene locus within the nuclear periphery with SEM from three independent replicates is shown. “*n*” represents the total number of FISH signals analyzed from all replicates. The *CA* gene locus distribution data under normal conditions were compared with those under excess copper conditions in WT and *crwn1crwn4*. Significance was determined using unpaired two-sided *t*-test.

We formulated a hypothesis to explain how the nuclear lamina proteins regulate the expression of *CA* genes. The hypothesis was that the CRWNs change the spatial arrangement of the *CA* genes in the nucleus through interacting with them, which affects their transcriptional activity. To test this hypothesis, we investigated the interaction between CRWNs and *CA* genes. Chromatin immunoprecipitation (ChIP) assays using CRWN1-EYFP and anti-GFP antibody failed to identify CRWN1-binding sites. Therefore, we used chromatin integration labeling (ChIL) assay^25^, which is based on an antibody reaction like ChIP but does not require immunoprecipitation. First, we prepared *pCRWN1*::CRWN1-EYFP in *crwn1* and *p35S*::GFP in WT for the ChIL assay. CRWN1-EYFP and CRWN1-binding DNA were cross-linked with formaldehyde and then primary and secondary antibody reactions were performed to label the CRWN1 (Fig. 5a). The secondary antibody was conjugated with oligo DNA fragments containing the T7 promoter and Tn5 transposase target sequences. After integration between CRWN1-binding DNA and oligo DNA fragments, RNAs were synthesized from CRWN1-binding DNA by T7 RNA polymerase in vitro. Complementary DNA (cDNA) libraries were constructed from purified RNAs. First, we confirmed that CRWN1-binding sites can be detected by ChIL assay. The possible CRWN1-binding sites were detected by qPCR using the primer pairs designed from sequences within the PR1 promoter and pericentromeric region because it was suggested that a protein complex of CRWN1 and NLT9, which is a gene involved in immune responses, bound to the PR1 promoter under normal conditions^21^ and CRWN1 interacted with centromeric and pericentromeric regions^26^. The PR1 promoter and pericentromeric region were significantly accumulated in CRWN1 ChILed DNA compared with the level in the control, which suggested that the ChIL assay can reveal the CRWN1-binding sites (Supplementary Fig. 6). CRWN1-binding sequences were detected by qPCR using 12 primer pairs designed from sequences within the cluster of *CA* genes (Fig. 5b). The values obtained in the CRWN1 ChIL-qPCR were normalized to those obtained in the GFP ChIL-qPCR. In the CRWN1 ChIL-qPCR analysis, specific enrichment of the *CA* gene locus was not detected under normal conditions, but the position 1 (p1), p3, p5, and p8 fragments were significantly enriched only under excess copper conditions (Fig. 5b). This suggested that the *CA* gene locus interacted with CRWN1 in a manner dependent on the amount of copper.

Next, we performed ChIP assay for histone H3, H4 acetylation (H4ac) as an epigenetic mark of transcriptionally active chromatin, and H3 lysine 27 trimethylation (H3K27me3) as a mark of inactive chromatin to evaluate the chromatin status on the *CA* gene locus. Under normal conditions, no differences were detected between WT and *crwn1crwn4*; however, under excess copper conditions, the amount of H3 was significantly reduced on p1, p5 and p7, and H4ac was increased on p2 and p3 in WT compared with the levels in *crwn1crwn4* (Fig. 5c, d). This suggested that histones were removed and chromatin was loosened in the *CA* locus to activate gene expression in response to the excess copper. H3K27me3 showed a similar pattern between WT and *crwn1crwn4* (Supplementary Fig. 7).

Finally, we determined whether the interaction between CRWNs and the *CA* gene occurred in the nuclear lamina. First, we checked the localization of CRWN1 under normal and excess copper conditions (Supplementary Fig. 8). CRWN1 was localized at the nuclear periphery under both conditions. We also checked the position of the *CA* gene locus by padlock FISH, which can visualize not only repetitive sequences but also a single gene locus in the nucleus^27^ (Fig. 5e and Supplementary Fig. 9a). We defined the nuclear periphery as the area 0.2 µm or closer to the nuclear edge, in accordance with a previously described method^27^. The percentage of the *CA* gene locus localized at the nuclear periphery was significantly increased from 22 ± 6% under normal conditions to 43 ± 5% under excess copper conditions in WT (Fig. 5d, e). Interestingly, the percentages of the *CA* gene locus at the nuclear periphery were similar under the two conditions in *crwn1crwn4*. In other words, the *CA* gene positioning was not altered in response to copper stress in *crwn1crwn4*. These results suggested that CRWNs mediate the change in *CA* gene positioning through interacting with the gene locus.

## Discussion

Here, we show that CRWNs localize at the nuclear lamina and form interactions that build the meshwork structure. The *CA* gene locus, at which *CA* genes are tandemly localized, interacts with CRWN1 and shifts to the nuclear periphery when the genes are activated under excess copper conditions, indicating that CRWN-dependent gene positioning is associated with *CA* gene activity and copper tolerance. When excess copper ions enter the cell, they should be immediately chelated and discharged by cytoplasmic proteins or chemicals to protect the cytoplasm from copper toxicity. *CA* genes might be involved in the chelating of copper ions in a manner similar to that for other heavy metal-associated proteins^24^. In fact, excess copper ions activate the expression of *CA* genes (Fig. 4b, c).

Clustered genes like the *CA* genes are well known as operons in prokaryotes. The genes clustered together in an operon are co-regulated by a single promoter and operator and are co-transcribed into a single mRNA, which leads to the production of a polycistronic mRNA^28^. Operons have also been discovered in eukaryotes such as *Caenorhabditis elegans*^29^. Prokaryotic operons form a polycistronic mRNA, whereas *C. elegans* operons form a polycistronic pre-mRNA, which is subsequently divided into monocistronic mRNAs. Both operon types are transcribed from single promoters and produce polycistronic pre-mRNA or mature mRNA, resulting in the synchronized expression of the clustered genes. Operon-like gene clusters have also been reported in *A. thaliana*^30–32^. The genes in a cluster are coordinately expressed, although they each have a promoter and are not co-transcribed into a single pre-mRNA and mature mRNA; hence, the regulatory mechanisms of the coordinated expression were not revealed. In this study, the clustered *CA* genes were transcriptionally co-regulated in response to excess copper. We propose that nuclear positioning of the *CA* genes impacts on their transcriptional activity.

The positioning of genes is positively regulated and affects their expression levels during cell differentiation, maturation, and tissue development in animals^33–35^. Generally, heterochromatin is mainly localized at the nuclear periphery and euchromatin is in the nucleoplasm in animals. Similar tendencies were observed in plants, according to a study on the genomic regions bound to the nucleoporin NUP1, which is a subunit of the nuclear pore complex (NPC)^36^. A ChIP-seq analysis of NUP1 revealed that its binding regions are enriched in silenced genes, transposable elements, and heterochromatin. In addition, recently, CRWN1-binding regions that are similar to NUP1-binding regions were also reported^26^. Silenced biosynthetic gene clusters approach the nuclear periphery, while activated clusters relocate away from it^37^. Other studies found that chromocenters are specifically localized at the nuclear periphery^38^. These results suggest that the nuclear periphery was occupied by heterochromatic and inactive regions in plants. However, some reports suggest that the nuclear periphery can activate gene expression. The nucleoporin Seh1 conjugated with LacI and YFP (She1–LacI–YFP) and LacO fused to the luciferase reporter gene (LacO:Luc) were co-expressed in *A. thaliana* to investigate the effect of the positioning of genes on their expression^39^. She1–LacI–YFP was localized at the nuclear periphery. Through the strong interaction between LacI protein and LacO DNA sequence, LacO:Luc was probably located at the nuclear periphery and the expression level of Luc was elevated compared with that of the control plants expressing only LacO:Luc. A report has been published on gene repositioning in plants focusing on the chlorophyll a/b-binding protein (CAB) gene locus, where three CAB genes are tandemly located^27^. The locus of the CAB genes, which are involved in de-etiolation, moves from the nucleoplasm to the nuclear periphery in response to red and far-red light. The repositioning of this locus induces a dramatic activation of CAB genes. The repositioning of this locus does not occur in phytochrome signaling mutants, suggesting that light-dependent repositioning of the CAB gene locus is mediated by the phytochrome signaling pathway. However, the molecular mechanism of tethering to the nuclear periphery has not been elucidated. The activation of *CA* genes at the nuclear periphery is consistent with a previous report describing luciferase gene and CAB gene activation at the nuclear periphery. These results suggest that the nuclear periphery in plants could be divided into gene-activating and -suppressing regions.

The findings from the present study showed that the expression of the *CA7*, *CA8*, and *CA10* genes was upregulated whereas CRWN1 interacted with *CA3*, *CA5*, and *CA8* in response to excess copper in 9-day-old plants. Under excess copper conditions, the levels of histone H3 in *CA3*, *CA5*, and upstream of *CA8* were higher and that of H4ac in *CA5* was lower in *crwn1crwn4* than in WT (Fig. 5c, d). CRWN1-binding sites and the site where histone was acetylated and removed overlapped, suggesting that the acetylation and removal of histones in the gene locus may be required for the interaction between the *CA* gene locus and CRWN1, rather than activation of the *CA* genes.

Our study indicated that CRWNs could tether the *CA* genes to the nuclear periphery; however, no activated genes were present in the CRWN1-binding site and it has not been reported that CRWNs directly regulate gene expression. Other proteins are probably involved in regulating the transcriptional activity of the *CA* genes. In a previous study, the gene clusters involved in stamen development were identified in *A. thaliana*^40^. The clustered gene expression was consistently downregulated in the *hta9hta11* double mutant exhibiting mutations in two H2A.Z genes, but not in other mutants including *haf1* (histone acetyltransferase), *atx1* (H3K4 methyltransferase), and *ref6* (H3K27 demethylase). In the case of the *CA* genes, histone variants may be involved in the regulation of gene activation. It was also reported that transcription factors are closely related to gene regulation in a manner dependent on gene positioning in yeast and animal cells^2^. The putative transcription factor bZIP18 was shown to interact with plant-specific nuclear envelope-associated proteins (NEAPs) in *A. thaliana*^41^. SUNs also bind to both NEAPs and CRWNs, suggesting that CRWNs indirectly interact with bZIP18^42^. Therefore, bZIP18 may regulate transcriptional activity at the nuclear periphery.

Transcriptome analysis of *crwn1crwn4* revealed that CRWNs play roles in gene regulation. Among the DEGs, similar numbers of up- and downregulated genes were identified, suggesting that CRWNs are involved in both enhancing and silencing genetic pathways. For example, the *CA* genes were inhibited in *crwn1crwn4* in our study, whereas the expression of a gene involved in immune responses, *PR1*, was found to be enhanced in *crwn1crwn2* in a previous study^21^. The GO analysis showed that genes responsive to biotic and abiotic stimuli were predominantly up- and downregulated in *crwn1crwn4*, consistent with the results of a previous study showing that CRWNs regulate genes related to immune responses^21,43^. The GO data implied that CRWNs are involved in many environmental stress responses.

## Methods

### Plant material and growth conditions

*Arabidopsis thaliana* Columbia-0 was used as the wild type. The mutants *crwn1* to *crwn4*, *crwn1crwn4*, and *crwn2crwn3* were described as *linc1* to *linc4*, *linc1linc4*, and *linc2linc3* disruptants, respectively, in a previous study^18^. The *crwn1crwn2* mutants were made by crossing *crwn1* and *crwn2*. *A. thaliana* seeds were sterilized with sodium hypochlorite, vernalized at 4 °C for 1 day, and grown on ½ MS medium at 22 °C under a 16-h light/8-h dark photoperiod. The copper concentration in ½ MS medium was adjusted by adding copper (II) sulfate solution.

### Plasmid construction

To create the CRWN-expressing lines, DNA fragments including the CRWN genomic DNA sequences and 2 kbp upstream from the start codon were cloned into the pENTR1A entry vector (Invitrogen, Carlsbad, CA, USA). *pCRWN1*::CRWN1-EYFP and *pCRWN2*::CRWN2-EYFP were constructed using the pGWB540 binary vector and *pCRWN3*::CRWN3-sGFP was constructed using the pMM1 binary vector^44^. The vectors were introduced into each of the *crwn1–3* mutants by *Agrobacterium tumefaciens*-mediated transformation, and *pCRWN1*::CRWN1-GUS, *pCRWN2*::CRWN2-GUS, *pCRWN3*::CRWN3-GUS, and *pCRWN4*::CRWN4-GUS were each cloned into pGWB533 and introduced into WT. For the Co-IP assay, *p35S*::CRWN1–4-GFP were previously described^18^ and *p35S*::CRWN1–3-tagRFP were each cloned into the pGWB560 binary vector. *p35S*::CRWN4-tagRFP was constructed using the pSY1R binary vector in which GFP in the pSY1 binary vector is replaced with tagRFP^18^. To create lines overexpressing *CA* genes, the coding sequences of *CA5* to *CA10* were each cloned into the pENTER D-TOPO vector. *p35S*::*CA5* to *p35S*::*CA10* were constructed using the pGWB502 binary vector and introduced into *crwn1crwn4*. As a positive control, *pCRWN1*::CRWN1-EYFP was also introduced into *crwn1crwn4*. The sequences of the primers used in these experiments are shown in Supplementary Data 1.

### Bright-field and fluorescence microscopy

To visualize the subcellular localization of CRWNs, plants were stained with propidium iodide (10 μg/ml; Molecular Probes, Eugene, OR, USA) for 5 min. Confocal fluorescence images were obtained using a confocal laser scanning microscope (FV1200; Olympus, Tokyo, Japan) equipped with 405, 473, and 559 nm LD laser lines and a 100 × 1.40 N.A. oil immersion objective (UPlanSApo; Olympus), a 60 × 1.40 N.A. oil immersion objective (PlanApo; Olympus), a 40 × 1.30 N.A. oil immersion objective (UPlanFL; Olympus), and a 20 × 0.75 dry objective (UPlanSApo; Olympus). *pCRWN1*::CRWN1-GUS, *pCRWN2*::CRWN2-GUS, *pCRWN3*::CRWN3-GUS, and *pCRWN4*::CRWN4-GUS were fixed with 90% acetone on ice for 15 min. The fixed samples were washed with reaction buffer (50 mM phosphate buffer (pH 7.2), 2 mM K_3_Fe(CN)_6_, 2 mM K_4_Fe(CN)_6_, 0.1% NP-40) and were treated with 0.5 mM 5-bromo-4-chloro-3-indolyl-beta-D-glucuronide (X-Gluc) in reaction buffer at 37 °C for 16 h. After post fixation with 1% glutaraldehyde in reaction buffer for 2 h, the samples were treated with ethanol series (30, 50, 70, 90, 100%). Bright-field and fluorescence images were obtained using a BX51 microscope (Olympus). Image analysis was performed using ImageJ 1.51 g software (NIH, Bethesda, MD, USA).

### Immunoelectron microscopy

Immunoelectron microscopy was performed as described in a previous study^45^ with some modifications. *Arabidopsis* root tips were frozen in a high-pressure freezing machine (EM PACT; Leica Microsystems, Wetzlar, Germany). The samples were freeze-substituted with 0.25% glutaraldehyde and 0.1% uranyl acetate in 100% acetone at −80 °C for 4 days, and then gradually warmed (EM AFS; Leica Microsystems). The samples were washed with 100% acetone, infiltrated with methanol, and then embedded in LR White resin (London Resin Company Ltd., London, UK). Ultrathin sections (70–80 nm) on formvar-coated nickel grids were labeled with anti-GFP antibody (1:50, A11122; Thermo Fisher Scientific, Waltham, MA, USA) in 50 mM Tris-buffered saline (TBS). After washing with TBS, sections were labeled with 12-nm colloidal gold particles coupled to goat anti-rabbit IgG (1:20, AB_2338016; Jackson ImmunoResearch, West Grove, PA, USA). The sections were stained with 4% uranyl acetate for 20 min and then examined under a transmission electron microscope (JEM-1400; JEOL, Tokyo, Japan) at 80 kV.

### Immunofluorescence staining

Seven-day-old seedlings expressing *pCRWN1*::CRWN1-EYFP, *pCRWN2*::CRWN2-EYFP, *pCRWN3*::CRWN3-sGFP, and *p35S*::sGFP were fixed with 4% formaldehyde in phosphate-buffered saline (PBS) at 25 °C for 60 min and washed with PBS twice. After wiping the moisture off the samples, seedlings were chopped in chopping buffer (15 mM Tris–HCl pH 7.5, 2 mM EDTA, 0.5 mM spermine-4HCl, 80 mM KCl, 20 mM NaCl, and 0.1% Triton X-100) with a razor blade on a glass slide. The chopped sample was suspended in four volumes of nuclei suspension buffer (100 mM Tris–HCl pH 7.5, 50 mM KCl, 2 mM MgCl_2_, 5% sucrose, and 0.05% Tween-20) and then the solution was filtered through a 30-µm nylon mesh. A drop of the solution was placed on a cover slip and then allowed to dry overnight. After washing with 0.5% Triton X-100 in PBS, the nuclei were treated with 4% bovine serum albumin (BSA) in PBS at 25 °C for 30 min. The nuclei were then treated with anti-GFP antibody at 1:3000 (ab290; Abcam, Cambridge, UK) and 4 °C for 16 h. After washing with PBSt (0.05% Tween-20 in PBS) and blocking with 4% BSA in PBS, the nuclei were incubated with Alexa Fluor 488-conjugated anti-rabbit antibody at 1:1000 (A21206; Thermo Fisher Scientific) and 25 °C for 1 h. After washing with PBSt and pure water, the nuclei were mounted with VECTASHIELD Mounting Medium (H-1000; Vector Laboratories, Burlingame, CA, USA) and observed by a confocal microscope.

### Super-resolution microscopy

The nuclei isolated from the plants expressing *pCRWN1*::CRWN1-EYFP, *pCRWN2*::CRWN2-EYFP, *pCRWN3*::CRWN3-sGFP, and *p35S*::sGFP were placed on a cover slip and immunostained using anti-GFP antibody (ab290; Abcam) at 1:3000 and Alexa Fluor 488-conjugated anti-rabbit antibody at 1:1000, as mentioned above. The nuclei were mounted with ProLong™ Glass Antifade Mountant (P36982; Thermo Fisher Scientific) and stored at 4 °C. Super-resolution images were obtained using a STED microscope (SP8-gSTED; Leica Microsystems, Mannheim, Germany) equipped with a white light laser, a 592 nm STED laser, and a 100 × 1.40 N.A. oil immersion objective (HC PL APO CS2). The excitation wavelength at 488 nm and fluorescent signal in the range of 495–585 nm were detected by a HyD detector with 1.5–9.0-ns time gating. Images were obtained with 4 × line averaging and 2 × frame accumulation. The pixel size was set to 14 nm per pixel. Image processing was performed by deconvolution software (Huygens Professional ver.18.10.0p8 64b; Scientific Volume Imaging) with the default conditions. Images were further processed using ImageJ 1.51 g.

### Co-immunoprecipitation assay

Fluorescent protein-conjugated CRWNs were transiently expressed in *Nicotiana benthamiana* leaves by *Agrobacterium* infiltration. The leaves were sampled at 4 days after inoculation. Immunoprecipitation was performed with a µMACS GFP Isolation Kit (Miltenyi Biotec, Auburn, CA, USA). The leaves (1.0–2.0 g) were homogenized in µMACS lysis buffer (2.0–4.0 ml) containing Protease Inhibitor Cocktail Complete (Roche) and then the extracts were centrifuged at 10,000 × *g* for 10 min to obtain the soluble lysate. Anti-GFP antibody-conjugated magnetic beads were added to the lysate and the mixture was incubated at 4 °C for 30 min with gentle shaking. The GFP-conjugated proteins were isolated using a magnetic column, in accordance with the manufacturer’s instructions. The purified proteins were analyzed by western blotting using an anti-GFP antibody at 1:2000 (ab290; Abcam), an anti-tagRFP antibody at 1:500 (R10367; Thermo Fisher Scientific), and HRP conjugated anti-rabbit antibody at 1:10000 (458; MBL, Aichi, Japan)

### Yeast two-hybrid assay

The coding sequences of CRWN1–4 were each cloned into pGADT7 and pGBKT7. Y2HGold Yeast strain (Takara Bio, Shiga, Japan) was transformed using Frozen-EZ Yeast Transformation II (Zymo Research, Irvine, CA, USA). Transformants were selected on SD/-Leu/-Trip medium. The protein interactions were analyzed on SD/-Leu/-Trp/-His medium.

### Fluorescence in situ hybridization

Flower buds were sampled from 5- to 6-week-old plants and fixed in Farmer’s solution (acetic acid:ethanol, 1:3) for 1 h at 25 °C. Fixed buds were washed with 70% ethanol and then with distilled water for 5 min. The buds were treated with an enzyme solution (2% w/v cellulose Onozuka RS, 0.5% w/v pectolyase Y-23, 10 mM citrate buffer, pH 4.5) at 37 °C for 1 h. The buds were broken by pipetting and then filtered through a 100-µm nylon mesh. The filtered nuclear solution was centrifuged at 5000 × *g* for 1 min and the pellet was resuspended in Farmer’s solution. A drop of the nuclear solution was placed on a glass slide and allowed to dry. Then, FISH was performed as previously described^46^. A centromere probe was amplified by PCR and labeled by nick translation using DIG-Nick Translation Mix (Sigma-Aldrich, St. Louis, MO, USA). Hybridized probes were visualized using a rhodamine-conjugated anti-digoxigenin antibody (Sigma-Aldrich). The number, size, and fluorescence intensity of FISH signals were measured using ImageJ 1.51 g. A binary image was generated from the fluorescent image and “Analyze Particles” in ImageJ was used to determine the signal size. To measure the fluorescence intensity, one pericentromeric signal was clipped from the raw image and “Measure” was used to determine the signal intensity. Subsequently, the background was subtracted from the signal intensity.

### RNA sequencing

Total RNA was extracted from 14-day-old seedlings using an RNeasy Plant Mini Kit (Qiagen, Hilden, Germany) and treated with DNase I. Then, RNA-seq libraries were constructed using the TruSeq RNA library prep kit v2. The libraries were sequenced using a Nextseq 500 sequencer (Illumina, San Diego, CA, USA). Six independent biological replicates were analyzed for each genotype. Sequenced reads were mapped onto cDNA sequences of TAIR10 using Bowtie with –all –best –strata settings. Normalization and DEG detection were performed using the R package TCC ver. 1.28.0^47^. The criteria for DEGs were as follows: fold value > 1.5 or <0.667 and FDR < 0.05. The GO analysis was performed using the PANTHER (Released 20171205).

### Chromatin integration labeling method

A ChIL assay was performed as described previously with some modifications^25^. Twenty to thirty 9-day-old seedlings expressing *pCRWN1*::CRWN1-EYFP and *p35S*::GFP were fixed with fixation buffer (1% formaldehyde in PBS) for 30 min under a vacuum and then 2 M glycine was added (final concentration, 150 mM). After washing with PBS, the seedlings were drained on a paper towel and then chopped in 50 µl of chopping buffer with a razor blade on a glass slide. The chopped sample was suspended in four volumes of nuclei suspension buffer (100 mM Tris–HCl pH 7.5, 50 mM KCl, 2 mM MgCl_2_, 5% sucrose, and 0.05% Tween-20) and then the solution was filtered through a 30-µm nylon mesh. Drops of the solution were added to wells of a 96-well plate and allowed to dry for several hours. The plate was either used immediately or stored at −80 °C. After washing with PBS, the nuclei were treated with 0.5% Triton X-100 and then with Blocking One P (Nacalai Tesque, Kyoto, Japan) for 20 min at 25 °C. After washing, anti-GFP antibody (ab290; Abcam) diluted 1:3000 in 0.1x Blocking One P in PBS was added and the plate was incubated for 6 h at 25 °C. After washing, secondary antibody solution (2 µg/ml oligo-conjugated anti-rabbit antibody, 0.1x Blocking One P, and 0.5 M NaCl in PBS) was added and the plate was incubated at 4 °C for 16 h. The nuclei were washed with ice-cold PBS and then treated with 1.77 µg/ml Tn5 transposase in dialysis solution (0.1 M NaCl, 0.1 mM EDTA, 1 mM DTT, 0.1% Triton X-100, 10% glycerol, 50 mM HEPES-KOH, pH 7.2) and then 2 nM Tn5-MEDS-B oligo in dialysis solution was added. After washing with PBS and dialysis solution, the nuclei were treated with TAPS-DMF buffer (5 mM MgCl_2_, 10% DMF, 10 mM TAPS-NaOH, pH 8.5) at 37 °C for 1 h and then the supernatant was removed and 0.2% sodium dodecyl sulfate (SDS) was added. After washing with T4 DNA ligase reaction buffer, the nuclei were treated with fill-in solution [200 U T4 ligase (New England Biolabs, Ipswich, MA, USA), 1.5 U T4 DNA polymerase (New England Biolabs), and 0.1 mM dNTP mix in T4 DNA ligase reaction buffer] at 25 °C for 30 min and then 0.2% (w/v) SDS was added. After washing with PBS and T7 RNA polymerase buffer, the nuclei were treated with in vitro translation solution [1000 U T7 RNA polymerase (TRL-252; Toyobo, Osaka, Japan), 40 U RNase inhibitor (Toyobo), 2 mM rNTPs in T7 RNA polymerase buffer] at 37 °C for 16 h and then 7 U DNase I (Nippon Gene, Tokyo, Japan) was added. The synthesized RNA was purified using an RNeasy MinElute Cleanup Kit (Qiagen) and cDNA was synthesized from purified RNA using a Verso cDNA Synthesis Kit (Thermo Fisher).

### Chromatin immunoprecipitation

A ChIP assay was performed as described previously^48^. One gram of 9-day-old seedlings was frozen with liquid nitrogen, ground into fine powder with mortar and pestle, cross-linked in the nuclei isolation buffer (1% formaldehyde, 0.6% Triton X-100, 14.4 mM 2-mercaptoethanol,) with 1 mM Pefabloc SC (Merck, Hamburg, Germany) and complete protease inhibitor cocktail (Merck). M220 focused ultrasonicators (Covaris, Woburn, MA, USA) and milliTUBE 1 ml AFA Fiber (Covaris) were used for sonication. Sonicated samples were incubated with the antibody at 4 °C for overnight. Anti-histone H3 (ab1791; Abcam), anti-H4ac (06-866; Merck), and anti-H3K27me3 (07-449; Merck) were used. The samples were incubated with Protein G Magnetic Dynabeads (Thermo Fisher Scientific) at 4 °C for 2 h and then washed with low-salt RIPA buffer (50 mM Tris⋅HCl, pH 7.8, 150 mM NaCl, 1 mM EDTA, 1% Triton X-100, 0.1% SDS, 0.1% Sodium deoxycholate and 1% Complete protease inhibitor (Roche)), twice with high-salt RIPA buffer (50 mM Tris⋅HCl, pH 7.8, 500 mM NaCl, 1 mM EDTA, 1% Triton X-100, 0.1% SDS, 0.1% Sodium deoxycholate and 1% Complete protease inhibitor (Roche)), with LNDET buffer (250 mM LiCl, 1% IGEPAL, 1% Sodium deoxycholate, 1 mM EDTA, 10 mM Tris–HCl pH 7.8), and with TE buffer. After the elution buffer (10 mM Tris–HCl pH 7.8, 0.3 M NaCl, 5 mM EDTA, 0.5% SDS) was added to the beads, the beads were incubated overnight at 65 °C. The lysis was treated with 200 ng/ml RNaseA at 37 °C for 30 min and then treated with 800 ng/ml Proteinase K and 400 ng/ml glycogen at 37 °C for 2 h. After phenol chloroform extraction and ethanol precipitation, the pellet was suspended in Buffer EB (Qiagen). Collected DNA was used for real time PCR.

### Padlock FISH

Twenty to thirty 9-day-old seedlings were fixed with fixation buffer (4% formaldehyde, 10 mM Tris–HCl pH 7.5, 10 mM EDTA, and 100 mM NaCl) for 20 min under a vacuum and then 2 M glycine was added (final concentration, 150 mM). The seedlings were drained on a paper towel and then chopped in 50 µl of chopping buffer with a razor blade on a glass slide. The chopped sample was suspended in four volumes of nuclei suspension buffer and then the solution was filtered through a 30-µm nylon mesh. Drops of the solution were placed on a cover slip and allowed to dry overnight. The cover slips were either used immediately or stored at −20 °C. Padlock FISH was performed as previously described^27^ with some modifications and performed in a 55-μl SecureSeal chamber (Grace Bio-Labs, Bend, OR). After washing with 0.2% Triton X-100 in PBS, the samples were treated with 0.5 U/μl *Eco*RI at 37 °C for 30 min. After washing twice with buffer A (100 mM Tris–HCl pH 7.5, 150 mM NaCl and 0.05% Tween-20), the samples were treated with 0.2 U/μl of Lambda exonuclease (New England Biolabs) in Lambda exonuclease reaction buffer containing 0.2 μg/μl BSA and 10% glycerol at 37 °C for 30 min. After washing twice with buffer A, the nuclei were incubated with 0.1 μΜ Padlock probe in 2x SSC containing 20% formamide and 0.5 μg/μl sonicated salmon sperm at 37 °C for 15 min. The slides were washed with buffer B (2x SSC, 0.05% Tween-20) and buffer A and then treated with 0.1 U/μl T4 ligase (New England Biolabs) in T4 DNA ligase reaction buffer supplemented with 250 mM NaCl, 0.2 μg/μl BSA, and 10% glycerol at 37 °C for 15 min. Slides were washed in buffer B at 37 °C for 5 min, rinsed in buffer A and dehydrated in a series of 70, 85 and 100% ethanol. The nuclei were treated with 1 U/μl phi29 DNA polymerase (Fermentas, Burlington, Canada) in phi29 DNA polymerase reaction buffer supplemented with 0.25 mM dNTPs, 0.2 μg/μl BSA and 10% glycerol at 37 °C for 1 h and then rinsed in buffer A. 250 nM fluorescence-labeled detection probe in a solution of 2x SSC and 20% formamide was added to the sample and incubated at 37 °C for 20 min. After washing five times with buffer A and three time with PBS, the samples were stained with 500 ng/ml 4′,6-diamidino-2-phenylindole (DAPI) for 20 min. After washing three time with water, samples were mounted in Prolong Gold antifade reagent (Thermo Fisher Scientific). The sequence of the padlock probe and detection probe are shown in Supplementary Data 1. The distance from the nuclear edge to the *CA* gene locus was quantified using ImageJ 1.51 g. In the young plants used here, nuclei from WT and *crwn1crwn4* were of comparable size (Supplementary Fig. 9b).

### Reporting summary

Further information on research design is available in the Nature Research Reporting Summary linked to this article.

## Supplementary information

Supplementary Information

Descriptions of Additional Supplementary Files

Supplementary Data 1

Reporting Summary

## Data Availability

RNA-seq data in this study have been deposited in DDBJ Sequence Read Archive under accession number DRA010732. The authors declare that all other data supporting the findings of this study are available within the manuscript and its supplementary files or are available from the corresponding author upon request. Source data are provided with this paper.

## Source data

Source Data
