# Supplementary material for: Subnuclear gene positioning through lamina association affects copper tolerance
Source: Nat Commun. 2020 Nov 20;11:5914. doi: 10.1038/s41467-020-19621-z (PMC7679404; doi:10.1038/s41467-020-19621-z)
Supplement: Supplementary file 1 — Supplementary Information [file 41467_2020_19621_MOESM1_ESM.pdf]

Supplementary information

Subnuclear gene positioning through lamina association affects copper tolerance

Yuki Sakamoto[1,2], Mayuko Sato[3], Yoshikatsu Sato[4], Akihito Harada[5], Takamasa Suzuki[6], Chieko Goto[7], Kentaro Tamura[8], Kiminori Toyooka[3], Hiroshi Kimura[9], Yasuyuki Ohkawa[5], Ikuko Hara-Nishimura[10], Shingo Takagi[2], Sachihiro Matsunaga[1,11,12]*

[1]Imaging Frontier Center, Organization for Research Advancement, Tokyo University of Science, 2641 Yamazaki, Noda, Chiba 278-8510, Japan

[2]Department of Biological Sciences, Graduate School of Science, Osaka University, 1-1 Machikaneyama-cho, Toyonaka, Osaka 560-0043, Japan

[3]RIKEN Center for Sustainable Resource Science, Yokohama 230-0045, Japan

[4]Institute of Transformative Bio-Molecules, Nagoya University, Chikusa, Nagoya 464-8601, Japan

[5]Division of Transcriptomics, Medical Institute of Bioregulation, Kyushu University, 3-1-1 Maidashi, Higashi, Fukuoka 812-0054, Japan

[6]College of Bioscience and Biotechnology, Chubu University, Kasugai 487-8501, Japan

[7]Graduate School of Agricultural and Life Sciences, The University of Tokyo, 1-1-1 Yayoi, Bunkyo-ku, Tokyo 113-8657, Japan

[8]School of Food and Nutritional Sciences, University of Shizuoka, Shizuoka 422-8526, Japan

[9]Graduate School of Bioscience and Biotechnology, Tokyo Institute of Technology, Yokohama, Japan

[10]Faculty of Science and Engineering, Konan University, Kobe 658-8501, Japan

[11]Department of Applied Biological Science, Faculty of Science and Technology, Tokyo University of Science, 2641 Yamazaki, Noda, Chiba 278-8510, Japan

[12]Department of Integrated Biosciences, Graduate School of Frontier Sciences,

The University of Tokyo, 5-1-5 Kashiwanoha, Kashiwa, Chiba 277-8562, Japan

*Corresponding author: sachi@edu.k.u-tokyo.ac.jp

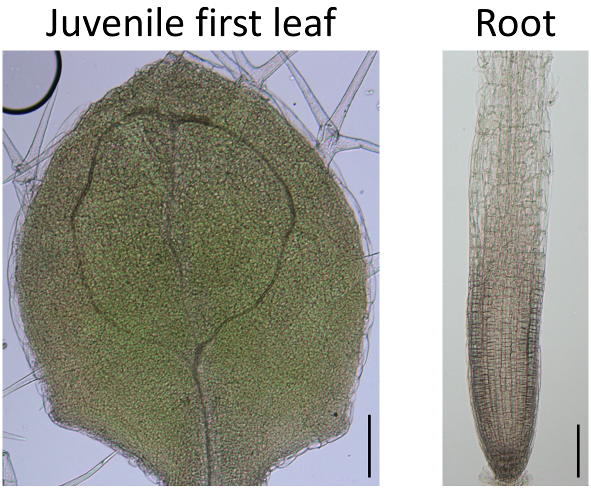

Supplementary Figure 1. CRWN4 expression pattern in *Arabidopsis thaliana*
GUS signals in 8-day-old seedlings of *pCRWN4*::CRWN4-GUS. Scale bars = 100 μm.

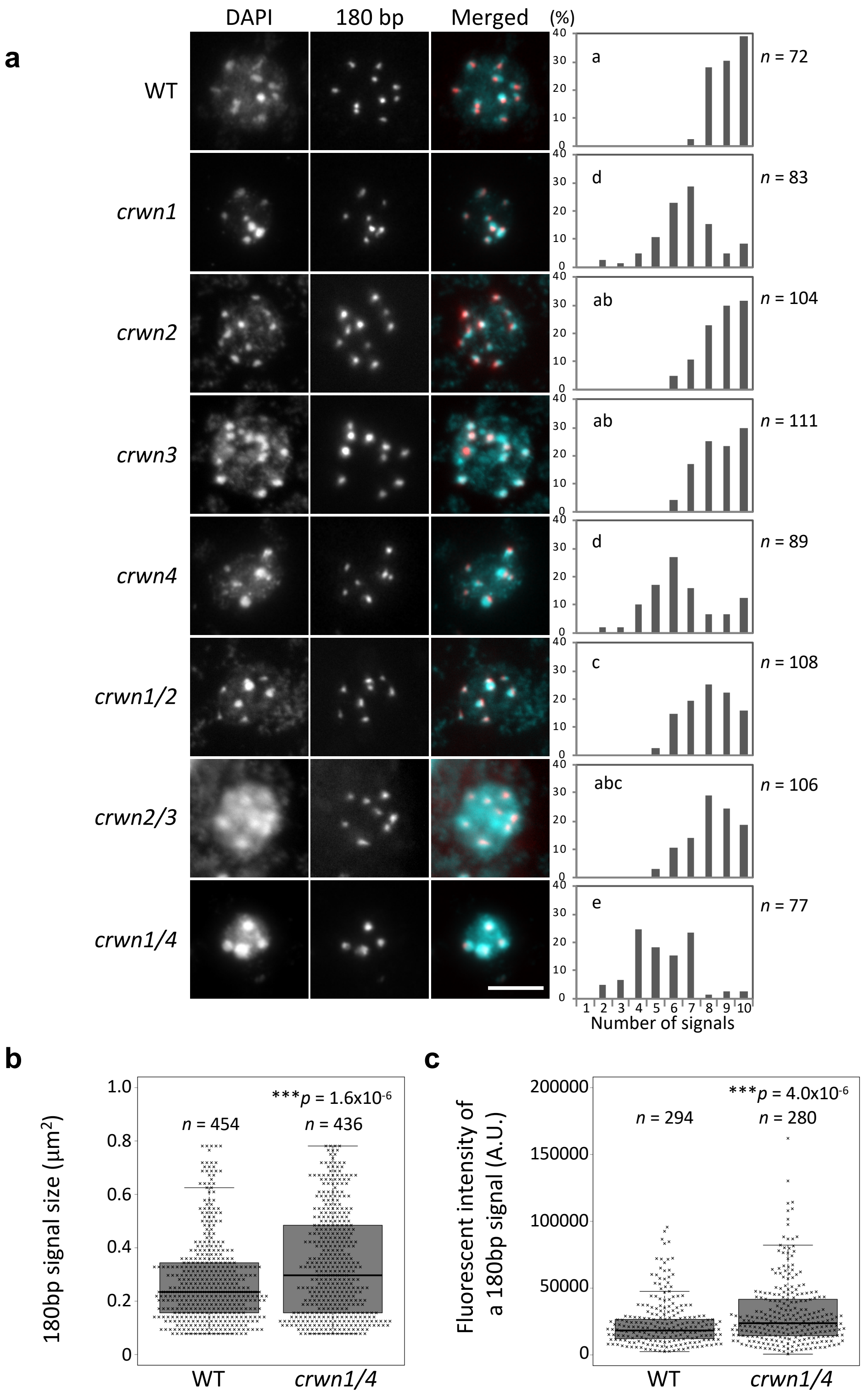

Supplementary Figure 2. CRWNs affected chromatin distribution pattern

a Fluorescent *in situ* hybridization (FISH) analyses of WT and *crwn* single and double mutants. The FISH probe is a 180-bp repeat enriched in the pericentromeric regions of all chromosomes. In the merged panels, FISH signals and DAPI fluorescence are red and cyan, respectively. Quantification data are represented as histograms in the right panels. Different letters indicate significant differences (chi-squared test, $p < 0.05$). The number of nuclei is indicated in the right side. **b, c** Box plots show the size (**b**) and fluorescence intensity (**c**) of FISH signal in WT and *crwn1crwn4*. Significance was determined using unpaired two-sided *t*-test. Boxplots are shown as median (middle bar), 25th and 75th percentiles (upper and lower limits of the box) and 1.5 × interquartile range (whiskers). Each data point represents a cross mark and the number of signals is indicated in the graph.

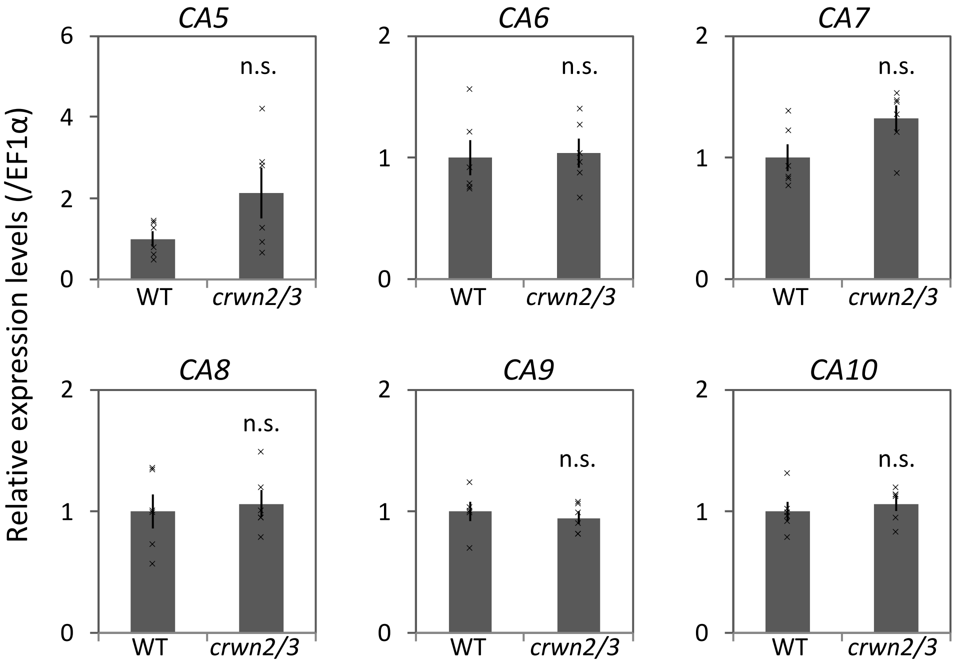

Supplementary Figure 3. Similar transcript levels of CA genes between *crwn2crwn3* and WT
CA gene transcript levels in 2-week-old WT and *crwn2crwn3* plants as determined by RT-PCR analysis. Data were normalized to EF1α mRNA levels and are expressed as mean ± SEM relative to the WT value (defined as 1). Significance was determined using unpaired two-sided *t*-test. (n ≥ 5 individual experiments). Each data point represents a cross mark.

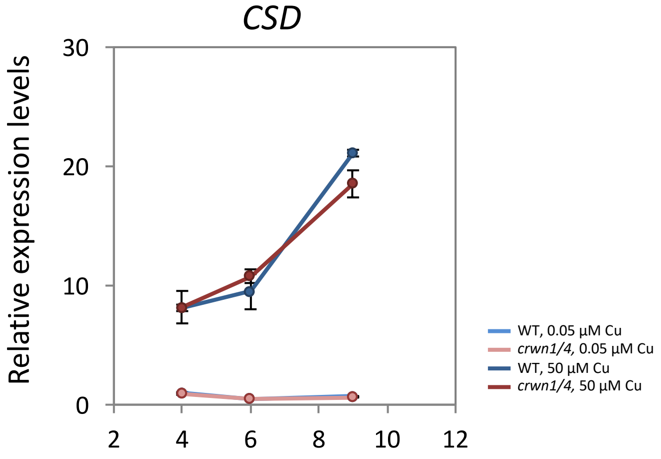

Supplementary Figure 4. Similar transcript levels of CSD genes between *crwn1crwn4* and WT
RT-PCR analysis of CSD gene transcript levels in 4-, 6-, and 9-day-old WT and *crwn1crwn4* under normal and excess copper conditions. Data were normalized to EF1α mRNA levels and are expressed as mean ± SEM relative to the value of 4-day-old WT under normal condition (defined as 1). Significance was determined using unpaired two-sided *t*-test (n ≥ 3 individual experiments).

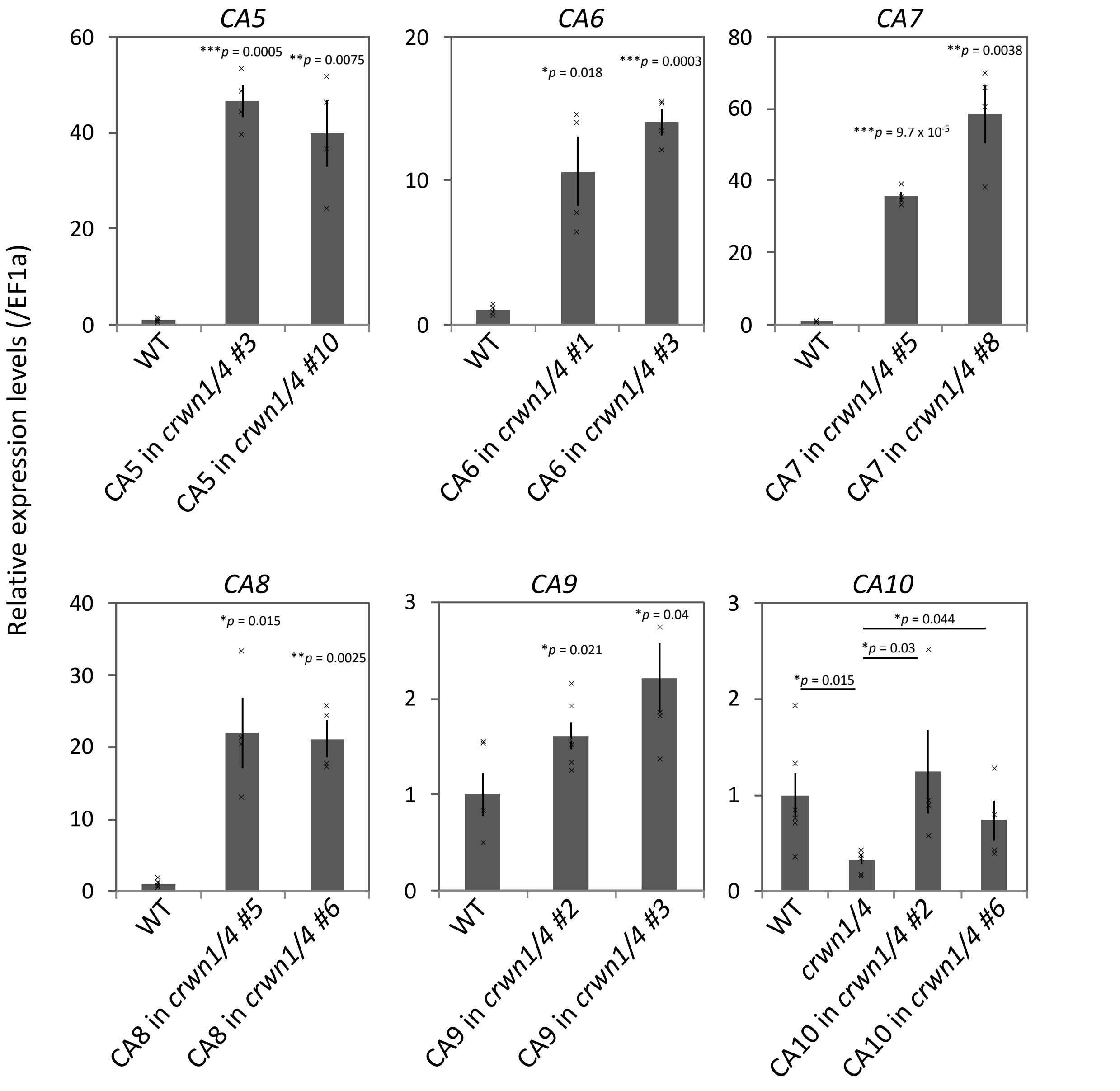

Supplementary Figure 5. Transformants overexpressing *CA* genes
CA gene transcript levels detected in 2-week-old plants as determined by RT-PCR analysis. Data were normalized to EF1α mRNA levels and are expressed as mean ± SEM relative to the value of 4-day-old WT under normal condition (defined as 1). Significance was determined using unpaired two-sided *t*-test ($n \geq 4$ individual experiments). Each data point represents a cross mark.

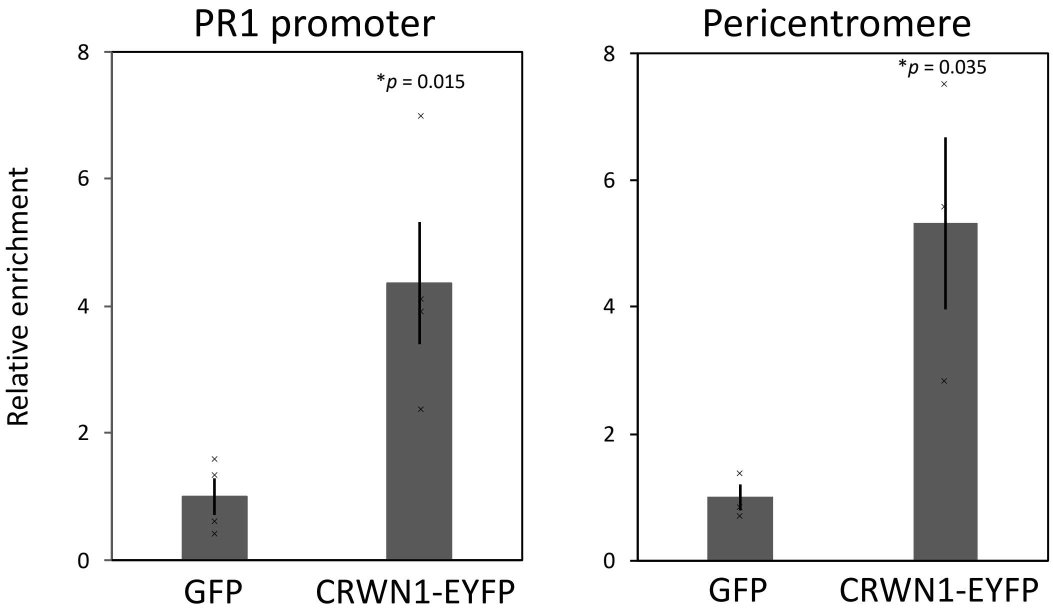

Supplementary Figure 6. CRWNs interact with PR1 promoter and pericentromeric region
ChIL-qPCR analyses using primer pairs designed within the PR1 promoter (n = 4 individual experiments) and pericentromeric region (n = 3 individual experiments) under normal conditions. Data are expressed as mean $\pm$ SEM relative to the GFP value (defined as 1). Significance was determined using unpaired two-sided t-test. Each data point represents a cross mark.

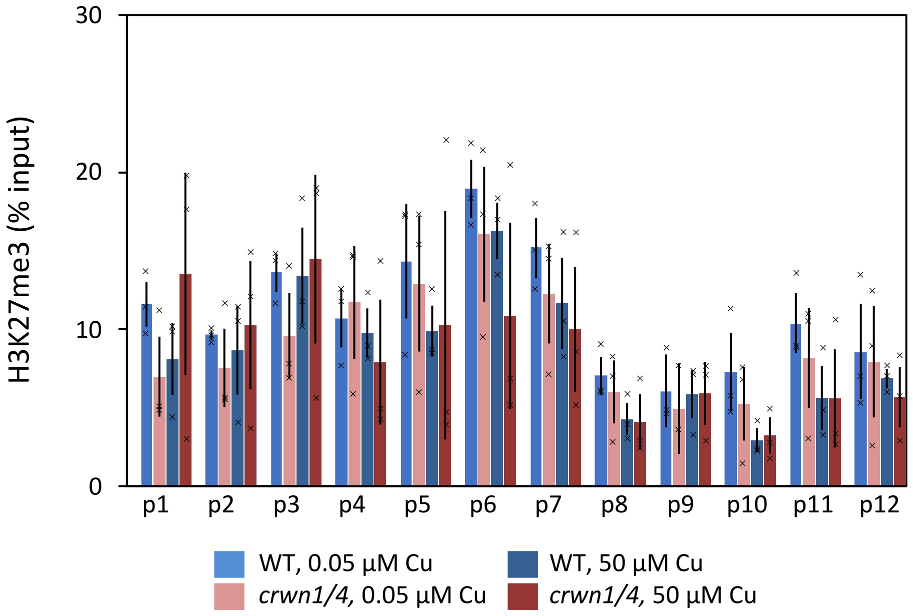

Supplementary Figure 7. H3K27me3 shows a similar distribution pattern between WT and *crwn1crwn4*

ChIP assay for H3 lysine 27 trimethylation in the *CA* gene locus under normal and excess copper conditions. Data are expressed as mean ± SEM. Significance was determined using unpaired two-sided *t*-test (n = 3 individual experiments). Each data point represents a cross mark.

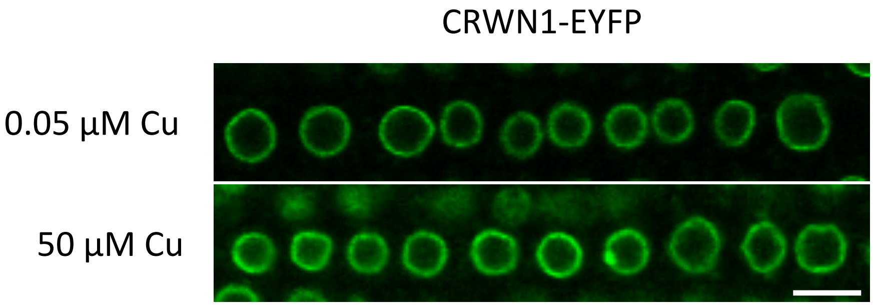

Supplementary Figure 8. CRWN1 localizes at the nuclear periphery under both normal and excess copper conditions

CRWN1-EYFP signals in root epidermal cells of 9-day-old seedlings grown under normal and excess copper conditions. Images show confocal optical section. Scale bar = 10 µm.

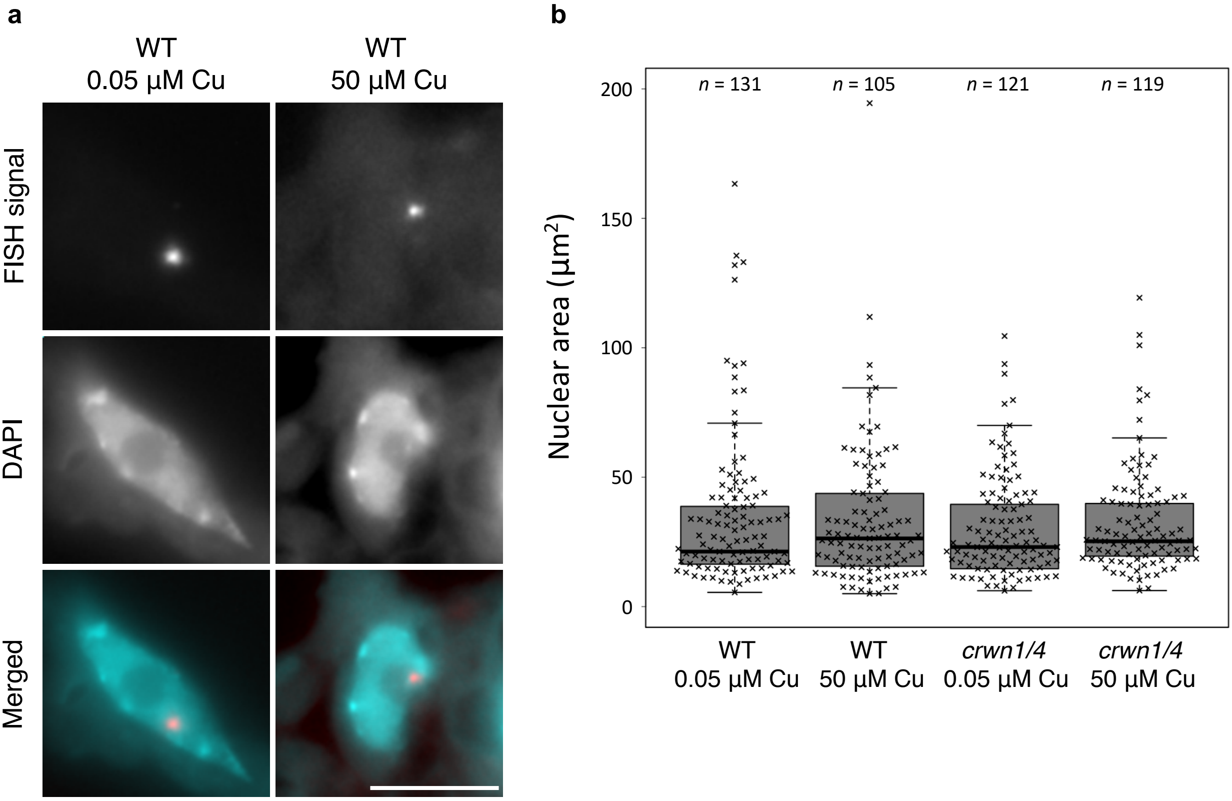

Supplementary Figure 9. There is no difference in the size of nuclei isolated from 9-day-old seedlings of WT and *crwn1crwn4*

a A FISH signal in an elongated nucleus was observed. Scale bar = 10 µm. Data are from single representative experiments that were reproduced three times. **b** Box plot shows the sizes of isolated nuclei used for padlock FISH measured from DAPI-stained images. Significant differences between WT and *crwn1crwn4* were not detected. Significance was determined using Tukey–Kramer method. Boxplots are shown as median (middle bar), 25th and 75th percentiles (upper and lower limits of the box) and 1.5 × interquartile range (whiskers). Each data point represents a cross mark and the number of samples is indicated in the graph.

AGI code	Gene name
AT5G52670	*Copper associated 1 (CA1)*
AT5G52680	*CA2*
AT5G52690	*CA3*
AT5G52700	*CA4*
AT5G52710	*CA5*
AT5G52720	*CA6*
AT5G52730	*CA7*
AT5G52740	*CA8*
AT5G52750	*CA9*
AT5G52760	*CA10*
AT5G52770	*CA11*

Supplementary Table 1. AGI codes and names of *CA* genes
