# Supplementary material for: Subnuclear gene positioning through lamina association affects copper tolerance
Source: Nat Commun. 2020 Nov 20;11:5914. doi: 10.1038/s41467-020-19621-z (PMC7679404; doi:10.1038/s41467-020-19621-z)
Supplement: Supplementary file 2 — Descriptions of Additional Supplementary Files [file 41467_2020_19621_MOESM2_ESM.pdf]

Descriptions of Additional Supplementary Files

Supplementary Data 1

Description: Primer and probe list
