# Supplementary material for: Subnuclear gene positioning through lamina association affects copper tolerance
Source: Nat Commun. 2020 Nov 20;11:5914. doi: 10.1038/s41467-020-19621-z (PMC7679404; doi:10.1038/s41467-020-19621-z)
Supplement: Supplementary file 4 — Reporting Summary [file 41467_2020_19621_MOESM4_ESM.pdf]

natureresearch

Corresponding author(s): Sachihiro Matsunaga

Last updated by author(s): Oct 15, 2020

Reporting Summary

Nature Research wishes to improve the reproducibility of the work that we publish. This form provides structure for consistency and transparency in reporting. For further information on Nature Research policies, see Authors & Referees and the Editorial Policy Checklist .

Statistics

For all statistical analyses, confirm that the following items are present in the figure legend, table legend, main text, or Methods section.

n/a	Confirmed	
☐	☒	The exact sample size (*n*) for each experimental group/condition, given as a discrete number and unit of measurement
☐	☒	A statement on whether measurements were taken from distinct samples or whether the same sample was measured repeatedly
☐	☒	The statistical test(s) used AND whether they are one- or two-sided<br>*Only common tests should be described solely by name; describe more complex techniques in the Methods section.*
☒	☐	A description of all covariates tested
☒	☐	A description of any assumptions or corrections, such as tests of normality and adjustment for multiple comparisons
☐	☒	A full description of the statistical parameters including central tendency (e.g. means) or other basic estimates (e.g. regression coefficient) AND variation (e.g. standard deviation) or associated estimates of uncertainty (e.g. confidence intervals)
☐	☒	For null hypothesis testing, the test statistic (e.g. *F*, *t*, *r*) with confidence intervals, effect sizes, degrees of freedom and *P* value noted<br>*Give P values as exact values whenever suitable.*
☒	☐	For Bayesian analysis, information on the choice of priors and Markov chain Monte Carlo settings
☒	☐	For hierarchical and complex designs, identification of the appropriate level for tests and full reporting of outcomes
☒	☐	Estimates of effect sizes (e.g. Cohen's *d*, Pearson's *r*), indicating how they were calculated

Our web collection on statistics for biologists contains articles on many of the points above.

Software and code

Policy information about availability of computer code

Data collection	We used ImageJ 1.51g (https://imagej.nih.gov/ij/) for the collection of imaging data.
Data analysis	We used TCC R package ver. 1.28.0 (http://bioconductor.org/packages/release/bioc/html/TCC.html) for RNA-seq analysis, PANTHER Overrepresentation Test (Released 20171205) (http://pantherdb.org/) for GO analysis, and Huygens Professional ver.18.10.0p8 64b for image processing.

For manuscripts utilizing custom algorithms or software that are central to the research but not yet described in published literature, software must be made available to editors/reviewers. We strongly encourage code deposition in a community repository (e.g. GitHub). See the Nature Research guidelines for submitting code & software for further information.

Data

Policy information about availability of data

All manuscripts must include a data availability statement. This statement should provide the following information, where applicable:

- Accession codes, unique identifiers, or web links for publicly available datasets
- A list of figures that have associated raw data
- A description of any restrictions on data availability

The authors declare that all data supporting the findings of this study are available within the article and its Supplementary Information files or are available from the corresponding author upon request.

Field-specific reporting

Please select the one below that is the best fit for your research. If you are not sure, read the appropriate sections before making your selection.

Life sciences study design

All studies must disclose on these points even when the disclosure is negative.

Sample size	We did not use statistical methods to determine sample size. In our study, sample size was determined to be adequate based on the magnitude and consistency of measurable differences between groups.
Data exclusions	No data were excluded.
Replication	Different amount of replicates were adopted by the analyses, according to the standards generally accepted in the plant research community.
Randomization	Plant materials were randomly picked up for our analyses and data collection.
Blinding	Not applicable.

Reporting for specific materials, systems and methods

We require information from authors about some types of materials, experimental systems and methods used in many studies. Here, indicate whether each material, system or method listed is relevant to your study. If you are not sure if a list item applies to your research, read the appropriate section before selecting a response.

Materials & experimental systems

n/a	Involved in the study
☐	☒ Antibodies
☒	☐ Eukaryotic cell lines
☒	☐ Palaeontology
☒	☐ Animals and other organisms
☒	☐ Human research participants
☒	☐ Clinical data

Methods

n/a	Involved in the study
☒	☐ ChIP-seq
☒	☐ Flow cytometry
☒	☐ MRI-based neuroimaging

Antibodies

Antibodies used

Rabbit polyclonal anti-GFP antibodies (A-11122, Thermo Fisher Scientific; ab290, Abcam)
Rabbit polyclonal anti-RFP antibody(R10367, Thermo Fisher Scientific)
12-nm colloidal gold particles coupled to goat anti-rabbit antibody (AB_2338016, Jackson ImmunoResearch)
Alexa Fluor 488-conjugated donkey anti-rabbit antibody (A21206, Thermo Fisher Scientific)
Goat polyclonal anti-rabbit pAb-HRP (458,MBL)
Rabbit polyclonal anti-H3 (ab1791; Abcam)
Rabbit polyclonal anti-H4ac (06-866; Merck)
Rabbit polyclonal anti-H3K27me3 (07-449; Merck)

Validation

Information of these antibodies are available in the web site.
A-11122, Thermo Fisher Scientific (https://www.thermofisher.com/antibody/product/GFP-Tag-Antibody-Polyclonal/A-11122)
ab290, Abcam (https://www.abcam.co.jp/gfp-antibody-chip-grade-ab290.html)
R10367, Thermo Fisher Scientific (https://www.thermofisher.com/antibody/product/RFP-Tag-Antibody-Polyclonal/R10367
AB_2338016, Jackson ImmunoResearch (https://www.jacksonimmuno.com/catalog/products/111-205-144)
A21206, Thermo Fisher Scientific (https://www.thermofisher.com/antibody/product/Donkey-anti-Rabbit-IgG-H-L-Highly-Cross-Adsorbed-Secondary-Antibody-Polyclonal/A-21206)
458, MBL (https://ruo.mbl.co.jp/bio/e/dtl/A/?pcd=458)
ab1791, Abcam (https://www.abcam.co.jp/histone-h3-antibody-nuclear-loading-control-and-chip-grade-ab1791.html)
06-866, Merck (https://www.merckmillipore.com/JP/ja/product/Anti-acetyl-Histone-H4-Antibody,MM_NF-06-866)
07-449, Merck (https://www.merckmillipore.com/JP/ja/product/Anti-trimethyl-Histone-H3-Lys27-Antibody,MM_NF-07-449)
